# Follicular fluid oxidative stress biomarkers and ART outcomes in PCOS women undergoing in vitro fertilization: A cross-sectional study

**DOI:** 10.18502/ijrm.v19i5.9254

**Published:** 2021-06-23

**Authors:** Kaivalya Gongadashetti, Pankush Gupta, Rima Dada, Neena Malhotra

**Affiliations:** ^1^Department of Obstetrics and Gynaecology, All India Institute of Medical Sciences, New Delhi, India.; ^2^Department of Anatomy, All India Institute of Medical Sciences, New Delhi, India.

**Keywords:** Oxidative stress, ART, PCOS, Infertility, 8-IP.

## Abstract

**Background:**

Polycystic ovarian syndrome (PCOS) is the most common cause of anovulatory infertility. Oxidative stress (OS), which plays an important role in determining the developmental competence of an oocyte, may be involved in understanding infertility and poor outcomes cycles in PCOS women undergoing in vitro fertilization (IVF).

**Objective:**

To measure OS biomarkers in the follicular fluid of PCOS women undergoing IVF.

**Materials and Methods:**

In this cross-sectional study, 100 women with PCOS (n = 43) and tubal factor (n = 57) undergoing IVF, who were referred to a tertiary medical center between January 2016 and September 2017 were enrolled. OS markers like reactive oxygen species (ROS), total antioxidant capacity (TAC), and 8-Isoprostane (8-IP) were tested in the follicular fluid and various IVF outcomes in the form of oocytes retrieved, fertilized, cleavage rate, grading of embryos and pregnancy outcomes were compared between the two groups.

**Results:**

The results indicated that the levels of ROS, TAC, and 8-IP were higher in the PCOS group compared to the tubal group (p = 0.21, p = 0.95, and p < 0.05, respectively). Biomarkers based on the number of retrieved oocytes, cleavage rate, and grades of embryos did not differ significantly in the two groups. The median ROS, TAC, and 8-IP were not significantly different in the two groups in relation to the pregnancies, although the 8-IP levels were significantly raised in the PCOS women who had a miscarriage (p = 0.02).

**Conclusion:**

This study concluded the possible role of OS in PCOS women with increased higher level of 8-IP biomarker as a potential biomarker that needs further evaluation.

## 1. Introduction

Polycystic ovary syndrome (PCOS) is the most common endocrinopathy, affecting about 6–10% women of reproductive age worldwide (1). PCOS forms a major cause of infertility with insulin resistance and hyperandrogenemia occupying a center stage in its pathogenesis (2). Insulin resistance increases the risk of metabolic syndrome in PCOS and associated cardiovascular morbidity. Increased glucose levels due to insulin resistance cause increased lipid peroxidation and generation of oxidants (3). There is a delicate balance of oxidants and antioxidants in oocyte and its surrounding environment including cumulus complexes and follicular fluid (FF), and any disruption in this balance can compromise oocyte competence (4). Insulin resistance and accompanying hyperglycemia cause increased oxidative stress (OS) in PCOS women affecting steroidogenesis thereby affecting follicular development (5). This altered milieu of oocyte due to OS has been associated with infertility in PCOS women (6). Measuring OS in PCOS can help in better understanding this syndrome and taking precautionary steps to reduce its burden. FF forms the immediate microenvironment of the developing oocyte and is the best medium to assess OS (7). A few studies on OS biomarkers in FF of non-PCOS women and assisted reproductive techniques (ART) outcomes have shown different results (8–10).

OS markers followed across various studies include measuring the reactive oxygen species (ROS) levels, total antioxidant capacity (TAC), and lipid peroxidation such as Malonaldehyde (MDA) levels in serum showing variable correlations (11–14) in PCOS females. Recently, 8-Isoprostane (8-IP) has been suggested as a specific, chemically stable, and quantitative marker of OS in vivo. Although, its possible role in seminal plasma has been explored (15), not much work has been done to evaluate this marker in FF and its role in oocyte development.

Considering the controversy and paucity of literature regarding OS markers in FF, we conducted this study on infertile females with PCOS undergoing in vitro fertilization (IVF). Further correlation of OS in FF with ART outcome was assessed.

## 2. Materials and Methods

In this cross-sectional study, 100 infertile women referred to the ART Department of All India Institute of Medical Sciences, New Delhi (India) for in vitro fertilization between January 2016 and September 2017 were evaluated in two PCOS (n = 43) and tubal factor (n = 57) groups. PCOS diagnosis was based on Rotterdam's criteria (16). All women with endometriosis, male factor, unexplained infertility, and diminished ovarian reserve [follicle-stimulating hormone > 10 mIU/ml, Anti-Müllerian hormone < 1 ng/ml, and antral follicle counts (AFCs) < 5] were excluded. All females were subjected to a pre-IVF workup in the form of complete blood count, liver and kidney function test, viral markers, electrocardiogram and chest X-ray (17). All PCOS females underwent GnRH antagonist protocol (Inj. Cetrorelix, Merck Serono, Mumbai, India) and tubal factor females were stimulated with different protocols based on their profile (18), and oocytes were retrieved. The fresh FF samples without any blood contamination were collected during oocyte retrieval and the levels of ROS and TAC were evaluated within 15–20 min of collection and stored at –80°C for determining 8-IP levels.

### Measurement of the ROS levels

Levels of ROS were measured in follicular fluid using luminol (5-amino-2,3, dihydro-l, 4-phthalazinedione; Sigma, Merck, St. Louis, MO, USA)-dependent chemiluminescence assay. A 400-μl of a well-mixed FF sample and 10 μl of luminol (5-Millimole) stock in DMSO (dimethyl sulfoxide) were added. ROS levels were assessed using the luminometer in the integrated mode for 15 min and results were expressed as counted photons per minute (cpm).

### Measurement of the TAC levels

TAC (millimole) was assessed using the commercially available kit (Cayman Chemical Item Number 709001, Ann Arbor, MI, USA) as per the specifications of the kit manufacturer described in Instruction manual.

### Measurement of the 8-IP levels 

8-IP is an OS marker indicative of lipid peroxidation. Its levels were estimated by commercially available Cayman's 8-IP EIA Kit (Cayman Chemical, Ann Arbor, MI, USA) using manufacturer's instruction manual.

### Embryo culture and transfer 

After oocytes were cultured and inseminated, fertilization was assessed after 16–18 hr. Fertilized oocytes were cultured, and on days 2 and 3, the cleavage of embryos was assessed and divided into two categories: those having a cleavage rate ≤ 95% and those with > 95%. The embryo quality was assessed on days 2, 3, and 5 post insemination and classified according to the alpha scoring system (19). The participants were categorized based on the grade of embryos. Those with grades 1 and 2 were considered in category A and grade 3 embryos were put in category B.

Embryo transfer (ET) was done on day 3 or 5 under ultrasound guidance using Cook's soft catheter (Cook Medical Bloomington, IN, USA). Luteal support was given with progesterone 100 mg daily intramuscular injections. Pregnancy was checked 14 days after ET with serum beta human chorionic gonadotrophin (HCG) levels and confirmation of clinical pregnancy was done with a viable fetal heart on a transvaginal scan, 6 wk after the ET.

### Ethical considerations

The study was approved by the Institutional Ethics Committee of All India Institute of Medical Sciences, New Delhi, India (IESC/T201/21.01.2015), and informed written consent was obtained from all participants prior to the study.

### Statistical analysis

Statistical analysis was carried out using Stata software 12.0 (College Station, Texas, USA). Data were presented as numbers, mean ± SD or median (min-max) as appropriate. Demographic and clinical characteristics and ART outcomes were compared between the PCOS and tubal groups using Fisher's Exact test (categorical variables), Student's *t* test (continuous variables with normal distribution), Wilcoxon rank-sum test, Mann–Whitney U-test (variables not following a normal distribution). Biomarkers ROS, TAC, and 8-IP between the PCOS and tubal groups were compared using the Wilcoxon rank-sum test. In addition, the correlation between the ART outcomes and biomarkers were calculated using Spearman's rank correlation coefficient. P-value < 0.05 was considered as statistically significant.

## 3. Results

Table I presents the demographic and biometric data of 100 participants. The median number of oocytes retrieved in the PCOS group was 13 compared to the tubal group where 9 oocytes were retrieved and the difference was significant (p < 0.05). Comparison of ART outcomes between the two groups like oocytes retrieved, fertilized, cleavage, embryo grades and pregnancy outcomes has been presented in Table II.

All FF samples were subjected to OS biomarkers measurement. Due to the staining of FF with blood and bursting of tubes during centrifugation, three of the PCOS and two of the tubal group samples had to be discarded leaving 40 samples in the PCOS group and 55 samples in the tubal group. The OS biomarkers levels in the PCOS and tubal groups are presented in Table III, the levels of 8-IP were found to be significantly higher in the PCOS than the tubal group (p = 0.004).

Based on the number of oocytes retrieved, women were divided into three subgroups (i) < 10 oocytes, (ii) 10–20 oocytes, and (iii) > 20 oocytes. The levels of biomarkers were not found to be statistically different across the three subgroups (ROS p = 0.68, TAC p = 0.37, and 8-IP p = 0.26) of the PCOS group. Tubal group also showed similar results (ROS p = 0.93, TAC p = 0.68, and 8-IP p = 0.55). Biomarker levels were no different when analyzed between the cleaved and non-cleaved embryos in the two groups. While assessing the grade, embryos from category A of the PCOS group had no difference in the biomarkers as compared to category B in the PCOS group. The results were reproducible in the tubal group as well (Table IV).

Pregnancy outcomes were divided into three categories: nonpregnant, miscarriage, and pregnancy > 20 wk. In the PCOS group, when biomarkers were compared across the outcomes, only levels of 8-IP were significantly higher in the miscarriage group than in the other outcomes (p = 0.021). In the tubal group, this difference in the biomarkers was nonsignificant across all outcomes (Table IV).

Correlations between the intermediate IVF outcomes like oocytes retrieved, fertilized, cleavage rate, grades of embryos, and the three OS biomarkers were assessed. There was a statistically significant correlation between grade-2 embryos with ROS levels in the PCOS group (p = 0.03), though no such correlation was seen with other grades and biomarkers. Also, no correlation between other intermediate outcomes and OS biomarkers was seen in the PCOS and tubal groups.

**Table 1:** Baseline parameters in PCOS and tubal groups


**Parameters **	**Group I (PCOS) (n = 43)**	**Group II (Tubal) (n = 57)**	**p-value**
**Age (yr)**	30.51 ± 3.82 (22-39)	32.16 ± 3.78 (23-40)	0.04*
**BMI (kg/m${}^{2}$)**	25.25 ± 4.1	25.78 ± 3.7	0.50*
**AFC Left**	8 (0-18)	5 (0-10)	< 0.05**
**AFC Right**	8 (0-18)	6 (3-12)	< 0.05**
**AMH (ng/ml) **	3.6 (1.3-12.6)	3.02 (0.6-12.6)	0.01*
Data presented as Mean/Median ± SD (Range), *Student *t* test, **Mann–Whitney U-test, SD: Standard deviation, BMI: Body mass index, AFC: Antral follicle count, AMH: Anti-Müllerian hormone			

**Table 2:** Oxidative stress biomarkers in PCOS and tubal groups


**ART outcome**		**Group I (PCOS) (n = 43)**	**Group II (Tubal) (n = 57)**	**p-value**
**Preovulatory follicles**		15 (0–40)	13 (3–28)	0.006${}^{*}$
**Oocytes retrieved**		13 (0–31)	9 (2–27)	0.006*
**Oocytes fertilized**		8 (0–19)	6 (1–17)	0.010*
**Cleavage rate**		100% (0–100%)	100% (38–100%)	0.412*
**Embryos**		8 (0–19)	5 (0–17)	0.002*
**Grade I**		6 (0–17)	3 (0–17)	0.006*
**Grade II**		1 (0–4)	1 (0–4)	0.149*
**Grade III**		0 (0–2)	0 (0–3)	0.612*
**Grade IV**		0 (0–6)	0 (0–0)	—
**Pregnancy outcome**				
****	**Non pregnant **	31	43	0.931*
****	**Pregnancy (> 20 wk) **	6	7	
****	**Miscarriage**	6	7	

**Table 3:** Oxidative stress biomarkers in PCOS and tubal groups


**Biomarkers**	**PCOS (n = 40)**	**Tubal (n = 55)**	**p-value**
**Luminometer**	43052.5 (16447–2152082)	32925 (3319–686282)	0.21
**ROS (cpm) **	71.75 (27.41–3586.8)	54.88 (5.53–1143.8)	0.21
**TAC (mM of Trolox)**	4.45 (0.91–13.39)	4.07 (0.73–15.5)	0.95
**8-IP (pg/µl)**	57.18 (15.5–271.8)	39.2 (8.2–446.3)	0.004
Data presented as Median (min–max), Wilcoxon rank-sum test, ROS: Reactive oxygen species, TAC: Total antioxidant capacity, 8-IP: 8 Isoprostanes, PCOS: Polycystic ovarian syndrome, cpm: Counted photons per minute, mM: Millimole, pg: Picogram, µl: Microliters			

**Table 4:** Comparison of ART outcomes with oxidative stress biomarkers in PCOS and tubal groups

**Parameter**		**PCOS group (n = 40)**			**p-value**	**TUBAL group (n = 55)**			**p-value**
**Oocytes retrieved**		**≤ 10 (n = 16)**	**11–20 (n = 16)**	**> 20 (n = 8)**	**≤ 10 (n = 30)**	**11–20 (n = 20)**	**> 20 (n = 5)**	****
****	**ROS (cpm)**	99.98 (24.4–1512.0)	54.19 (30.1–3586.8)	94.62 (35.1–1158.3)	0.685	53.6 (5.5–1143.8)	61.65 (27.1–991.8)	47.38 (42.5–264.8)	0.929
****	**TAC (mM of trolox) **	4.45 (0.9–13.3)	4.96 (1.64–9.5)	2.76 (1.6–5.6)	0.368	4.16 (0.7–10.3)	4.13 (1.8–10.5)	1.14 (0.86–15.5)	0.679
****	**8IP (pg/μl)**	97.41 (21.3–271.8)	60.8 (15.5– 221.5)	51.77 (21.1–141.9)	0.259	24.35 (10.2–446.3)	48.4 (8.2–354.1)	46.2 (9.5–102.5)	0.553
**Cleavage rate**		**≤ 95% (n = 12)**	**> 95% (n = 28)**	**p-value**	**≤ 95% (n = 11)**	**> 95% (n = 44)**	**p-value**
****	**ROS (cpm)**	61.15 (28.7–1512.1)	78.61 (27.4–3586.8)	0.767	94.08 (33.1–991.9)	52.9 (5.5–1143.8)	0.365
	**TAC (mM of trolox)**	5.27 (1.6–7.5)	3.14 (0.9–13.3)	0.114	4.97 (2.2–10.5)	3.87 (0.7–15.5)	0.251
****	**8IP (pg/μl)**	64.34 (21.1–187.6)	54.1 (15.5–271.8)	1.00	39.2 (8.2–271)	39.10 (9.5–446.3)	0.983
**Grades of embryos**		**I and II (n = 33)**	**III and IV (n = 7)**	**p-value**	**I and II (n = 49)**	**III and IV (n = 6)**	**p-value**
****	**ROS (cpm)**	87.27 (27.41–1512.1)	65.29 (39.58–3586.8)	0.789	56.46 (5.5–1143.8)	44.10 (31.5–1014.9)	0.543
	**TAC (mM of trolox)**	3.44 (0.9–13.3)	6.11 (1.6–9.5)	0.193	4.07 (0.84–15.5)	3.59 (0.7–5.5)	0.466
****	**8IP (pg/μl)**	56.1 (15.5–271.8)	58.27 (42.5–221.5)	0.423	43.7 (8.2–446.3)	32.15 (14.2–129.7)	0.978
**Pregnancy outcome**		**No pregnancy (n = 31)**	**Pregnancy > 20 wks (n = 5)**	**Miscarriage (n = 4)**	**p-value**	**No pregnancy (n = 41)**	**Pregnancy > 20 wks (n = 7)**	**Miscarriage (n = 7)**	**p-value**
****	**ROS (cpm)**	73.56 (27.6–3586.8)	97.36 (27.4–1512.0)	50.03 (28.7–197.5)	0.652	57 (5.53–1143.8)	42.49 (34.2–654.1)	48.41 (40.8–233.7)	0.824
****	**TAC (mM of trolox) **	3.96 (0.91–9.5)	6.59 (2.2–13.4)	6.7 (0.95–7.3)	0.299	3.71 (0.8–10.5)	4.97 (0.7–7.7)	4.75 (1.9–15.5)	0.590
****	**8IP (pg/μl)**	53.29 (15.5–271.8)	58.27 (21.3–181.3)	188.2 (187.6–221.5)	0.021	39.2 (8.2–446.3)	34.51 (22.9–102.5)	52.98 (9.5–95.5)	0.739
Data presented as Median (min–max), Wilcoxon rank-sum test, ROS: Reactive oxygen species, TAC: Total antioxidant capacity, 8-IP: 8 Isoprostanes, PCOS: Polycystic ovarian syndrome, cpm: Counted photons per minute, mM: Millimole, pg: Picogram, µl: Microliters									

## 4. Discussion

The role of OS in female reproductive physiology and pathophysiology of various causes of infertility has received increasing interest. Researchers over the years have shifted focus from embryo quality to oocyte quality to favorably optimize IVF outcomes. The FF serves as an excellent biological window that provides easy access to study the metabolic changes occurring in the immediate microenvironment of an oocyte. Various OS markers have been reported in the FF but data on OS in PCOS women undergoing IVF is very sparse. Therefore, the present study attempted to find the significance of FF ROS, TAC and 8-IP in relation to the various outcomes of an IVF cycle in women with PCOS and tubal-factor infertility.

In the present study, we found no significant difference in the median ROS values between the PCOS and tubal groups. When comparing amongst the oocytes retrieved, fertilized rate, cleavage rate, and different grades of embryos, no difference in median ROS values were found between the two groups. This was corroborated by earlier reports (9, 10, 20) through a significant negative correlation between ROS level in FF and embryo quality, documented by Jozwik and colleagues (20). Our study showed a positive correlation of ROS with grade-2 embryos in the PCOS group of women. However, no correlation was seen with other grades of embryos and ROS values, therefore association cannot be derived. When the pregnancy outcomes were compared, no difference was found in ROS levels between the pregnant and non-pregnant women in the PCOS and tubal groups. This finding is in contradiction to a previous study where low levels of FF ROS were shown to predict success in IVF (10).

When TAC levels were compared between the PCOS and tubal groups, no significant difference was seen as opposed to the results by Oyawoye and colleagues, who found oxidant–antioxidant balance to be related to the etiology of infertility and the presence of polycystic ovary morphology (12). Similar to ROS, no correlation of TAC levels with oocytes retrieved, fertilization, cleavage, grading of embryos, and pregnancy outcome was seen. Pasqualotto and colleagues (14) also came up with similar conclusions although such findings have been refuted by a few other studies (11, 12). We used enzyme immunoassay for TAC levels in our study which has not been used in the previous comparable studies which increase the authenticity of our results.

A novel OS marker, 8-IP in the FF was evaluated which is a prostaglandin F2-like compound. It is produced by peroxidation of arachidonic acid catalyzed by free radical (21). Even for the study of some neurodegenerative diseases like Alzheimer's and Down's syndrome, serum 8-IP has been considered a very sensitive marker of OS (22). Its formation is modulated by antioxidant status and the levels increase dramatically in response to an oxidant injury (23).

The median 8-IP values in the present study were higher in the PCOS group in a statistically significant fashion. The median log 8-IP values were not significantly different in each group when compared with the ovarian response viz oocytes retrieved, fertilized rate, cleavage rate, and different grades of embryos. However, it was seen that in the pregnant group, the median 8-IP was significantly more in the PCOS women who had an abortion. This correlation was not seen in the tubal group. Previous studies have not found any correlation between 8-IP and pregnancy outcomes in non-PCOS females (21, 24). This new finding could suggest the presence of markers in the oocyte which could hint at an increased risk of miscarriage in a woman with PCOS. Whether this relationship is a cause or effect needs further assessment.

This study raises some important study questions which need further clarification. One of them being the role of antioxidants in infertile PCOS females. Antioxidants have previously been shown to increase dominant follicle selection, cytoplasmic maturation of MII oocyte and improved embryo development (25). Another question is the need to measure 8-IP in IVF cycles and if raised, the need for special measures to prevent miscarriages.

##  Limitation

Our study suffered a few drawbacks, including a small sample size limited on account of substantive costs of conducting the biomarker testing. Heterogeneity in the IVF protocols could have also resulted in differences between the two groups. Various external factors like environmental pollution, smoking, heavy metals, pesticides can increase levels of oxidants in our body (26), which may have acted as possible confounders. Most studies have been limited as even dietary patterns can alter results of OS biomarkers (27), but this was obviated by using 8-IP which is a stable marker uninfluenced by such factors and did show a correlation to women with PCOS. 8-IP has emerged as a better OS biomarker in this study requiring further research.

## 5. Conclusion

We can conclude that OS may have a role in PCOS-related IVF outcome as demonstrated by higher levels of 8-IPs in this group, although the other markers did not show any significant association. Also, 8-IP may be evaluated further as a significant marker to predict pregnancy loss in PCOS women and help in their prognostication during ART treatment. Larger studies are required to explore the correlation of these OS markers with ovarian-response parameters in different infertility groups based on etiology.

##  Conflict of Interest 

The authors report no conflict of interest.
